# Aluminum-Doping Effects on the Electronic States of Graphene Nanoflake: Diffusion and Hydrogen Storage Mechanism

**DOI:** 10.3390/nano13142046

**Published:** 2023-07-11

**Authors:** Hiroto Tachikawa, Yoshiki Izumi, Tetsuji Iyama, Shigeaki Abe, Ikuya Watanabe

**Affiliations:** 1 Department of Applied Chemistry, Graduate School of Engineering, Hokkaido University, Sapporo 060-8628, Japan; 2 Department of Dental and Biomedical Materials Science, Graduate School of Biomedical Sciences, Nagasaki University, Nagasaki 852-8102, Japan

**Keywords:** H_2_ storage, Al-doping, diffusion path, diffusion barrier, absorption spectrum, spin density, aluminum addition

## Abstract

Graphene nanoflakes are widely utilized as high-performance molecular devices due to their chemical stability and light weight. In the present study, the interaction of aluminum species with graphene nanoflake (denoted as GR-Al) has been investigated using the density functional theory (DFT) method to elucidate the doping effects of Al metal on the electronic states of GR. The mechanisms of the diffusion of Al on GR surface and the hydrogen storage of GR-Al were also investigated in detail. The neutral, mono-, di-, and trivalent Al ions (expressed as Al, Al^+^, Al^2+^, and Al^3+^, respectively) were examined as the Al species. The DFT calculations showed that the charge transfer interaction between Al and GR plays an important role in the binding of Al species to GR. The diffusion path of Al on GR surface was determined: the barrier heights of Al diffusion were calculated to be 2.1–2.8 kcal mol^−1^, which are lower than Li^+^ on GR (7.2 kcal/mol). The possibility of using GR-Al for hydrogen storage was also discussed on the basis of the theoretical results.

## 1. Introduction

Carbon-based materials such as graphene nanoflakes (GRs) and carbon nanotubes (CNTs) have been widely used as molecular devices in recent years because of their light weight and chemical stability [1,2,3,4,5,6,7,8]. Doping metals and atoms into these carbon materials generates new electronic states near the Fermi level and can significantly change their electronic properties [9,10]. Therefore, an investigation of metal- or atom-doped GR systems is of interest in the development of new high-performance electronic devices [11,12,13,14].

Mao et al. [15] experimentally investigated single-transition metal (TM) atom-doped GR to elucidate the catalyzing electrochemical reactions. TM-GR enhanced oxygen reduction reactions due to the electron coupling effects. They found that the Ni atom is effective for the reaction. Promthong et al. calculated the interaction of TM-doped GR with CO and CO_2_ molecules [16]. As TMs, Sc, Ti, V, Cr, Mn, Fe, Co, Ni, Cu, or Zn were examined. They showed that the Cr atom is most effective as a doping metal in CO and CO_2_ storage. In addition, it was found that the adsorption of the CO and CO_2_ molecules on TM-doped GR slightly influences the electronic conductance of the TM-doped GR.

TM-doped GRs could be used as a toxic HCl gas sensor. Tang et. al. investigated the interaction of TM-GR with HCl molecules (TM = Ti, V, Cr, Mn, Fe, Co) [17]. The Fe-doped GR demonstrated a better adsorption capacity and higher stability, even at high temperatures.

Recently, both single TM atoms and metal clusters have been studied. The structural, electronic, and magnetic properties of Fe_13_ and Al_13_ nanoparticles adsorbed on mono-vacancy defective GR have been determined using the density functional theory (DFT) method with the generalized gradient approximation (GGA) [18]. The binding energies of Fe_13_ and Al_13_ to the defective GR were calculated to be −6.98 and −3.84 eV, respectively. These strong bindings were due to the hybridization of the nanoparticles with the sp^2^ dangling bonds of neighboring carbons near the vacancy.

**Scheme 1:**
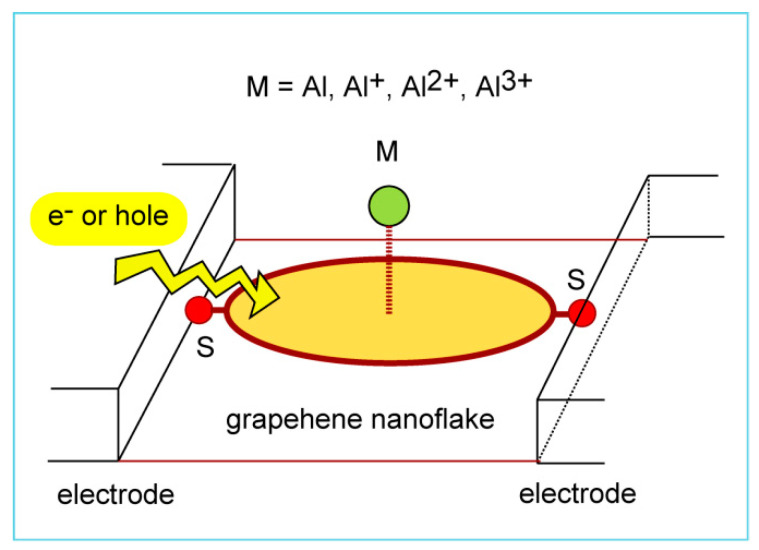
An example of the circuit board of the GR-Al system that can change the charge of Al.

The interaction between aluminum (Al) and GR is important to understanding the principles of Al-air batteries. However, there is little information on the interaction of GR with Al [19], although this knowledge is important for the development of high-performance aluminum ion batteries. Thus, the electronic states of TM-doped GRs are theoretically well understood. However, the interaction of Al with GR is not clearly understood. Al has specific valence charges, i.e., four valence charges (0, +1, +2 and +3). Therefore, it can be expected that several forms of interaction are generated by valence charges. However, there is no systematic theoretical work for this charge dependence. Using an external control of electron/hole injection to a GR-Al system, the valence charges of GR-Al can be arbitrarily hanged. Scheme 1 provides an example of the circuit board of the GR-Al system that can change the charge of Al. The charge of Al can be changed by externally injecting electrons or holes.

In the present study, the Al doping effects on the electronic states of GR were investigated using the DFT method. We focused our attention on changes in the electronic states of GR by the addition of charged Al. Also, the potential energy curve for the diffusion of Al on GR surface was investigated to elucidate the mechanism of the Al battery. Hydrogen (H_2_) is the ultimate clean energy source. To establish a hydrogen energy system, it is necessary to develop technologies for hydrogen storage and transportation, and to improve the social infrastructure [20,21,22]. In this study, the hydrogen storage ability of the GR-Al system is also investigated. Previously, hydrogen storage mechanisms have been studied for Li^+^, Na^+^, and Mg^2+^ on GR. Those metals have shown a strong hydrogen storage capacity. In this work, Al is examined as the metal atom and ions.

## 2. Method of Calculation

**Figure 1:**
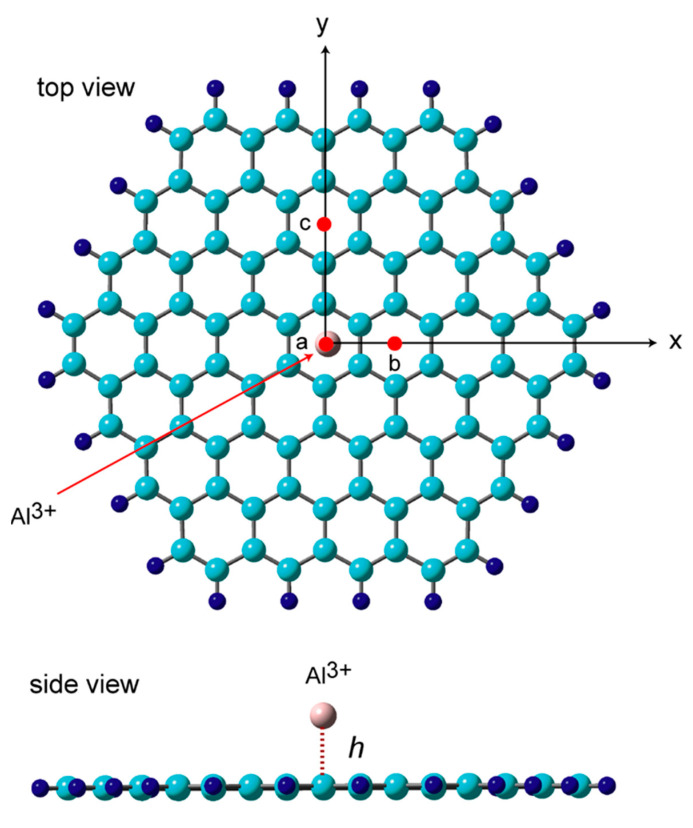
The optimized structure of GR37-Al^3+^ calculated at the CAM-B3LYP/6-311G(d,p) level. *h* means height of Al from GR surface: height is defined as vertical distance of Al from GR surface. Points a, b, and c mean hexagonal sites of Al on GR. x-axis and y-axis mean diffusion paths of Al on GR.

All DFT calculations were carried out using Gaussian 09 program package [23]. As a graphene nanoflake (GR), two polycyclic aromatic hydrocarbons composed of 19 and 37 benzene rings (denoted as GR19 and GR37, respectively) were utilized because these have symmetric structures. Figure 1 shows the structure of GR37 used in the present study.

The geometries of GR and GR-Al were fully optimized at the CAM-B3LYP/6-31G(d) and 6-311G(d,p) theory levels [24,25,26]. Previous works showed that these levels of theory provide reasonable electronic structures for graphene systems [27,28,29,30,31]. The electronic states of GR-Al were obtained by natural population analysis (NPA) and natural bond orbital (NBO) methods at the CAM-B3LYP/6-311G(d,p) level. The binding energy of Al to GR, E_bind_, is defined by

−E_bind_ = E(GR-Al) − [E(GR) + E(Al)] (1)

where E(GR-Al) means total energy of GR-Al, and E(GR) and E(Al) are total energies of GR and Al, respectively.

The diffusion barriers for Al diffusion on GR surface were calculated as follows: the heights of Al on GR37 changed from 2.0 to 2.8 Å, and then potential energy curves were calculated as a function of distance of Al from the center of GR37. The height (*h*) is defined as the vertical distance of Al from the GR surface.

For the H_2_ storage mechanism, a GR19-Al system was used and then the structures and electronic states of GR19-Al-(H_2_)_n_ (*n* = 1–8) were calculated at the CAM-B3LYP/6-31G(d) level. The binding energy of the nH_2_ molecules to GR-Al was defined as follows:

(2) $$-E_{bind}(n)=[E(GR-Al-(H_{2}{)}_{n})-(nE(H_{2})+E(GR-Al))]/n$$

The addition of H_2_ to GR-Al proceeds, and the H_2_ + GR-Al → H_2_-GR-Al addition reaction is exothermic when E_bind_(H_2_) is positive. Additional calculations were carried out at the same level of theory for Al^+^, Al^2+^, and Al^3+^ systems. We previously investigated interactions between graphene and various molecules using DFT at the same level of theory [27,28,29,30,31]. A similar technique was used for the GR-Al-(H_2_)_n_ system in this study.

## 3. Results

### 3.1. Structure of GR-Al

Figure 1 shows the optimized structure of GR37-Al^3+^ calculated at the CAM-B3LYP/6-311G(d,p) level. The Al^3+^ ion binds to the hexagonal site of the benzene ring of GR. The height of Al^3+^ was calculated to be 2.406 Å from the GR surface. Similar calculations were carried out for GR-Al, GR-Al^+^, and GR-Al^2+^. The heights of Al species on GR are summarized in Table 1. The heights for Al, Al^+^, Al^2+^, and Al^3+^ were 2.217, 2.273, 2.333, and 2.406 Å, respectively. The height increases with increasing Al charge. This is due to the fact that the positive charge of GR increases as the charge of Al is increased. Thus, the repulsive interaction between Al^+^ and GR^+^ becomes larger with larger valence charges.

The NPA charges of Al and GR are given in Table 1. In GR-Al(0), the charges were +0.864 for Al(0) and −0.864 for GR, indicating that the electron transfer occurred from Al to GR after the binding of Al to GR, i.e., charge transfer (CT) occurred between Al and GR, and the net charge of Al is changed to near mono-valent Al^+^ ion. In GR-Al with charges of +1, +2 and +3, the charges were calculated to be +0.931 (Al^+^), +0.924 (Al^2+^) and +0.912 (Al^3+^). These results indicated that the atomic charges oof Al were changed to near mono-valent Al^+^ in all cases. In case of GR-Al^+^, no electron transfer occurred. It should be noted that a large CT occurred in Al, Al^2+^ and Al^3+^ on GR.

Similar calculations were carried out for the GR19-Al system using the CAM-B3LYP/6-31G(d) method. The results are given in Table 2. Similar results were obtained.

### 3.2. Effects of Al Doping on Excitation Energies of Graphene

**Figure 2:**
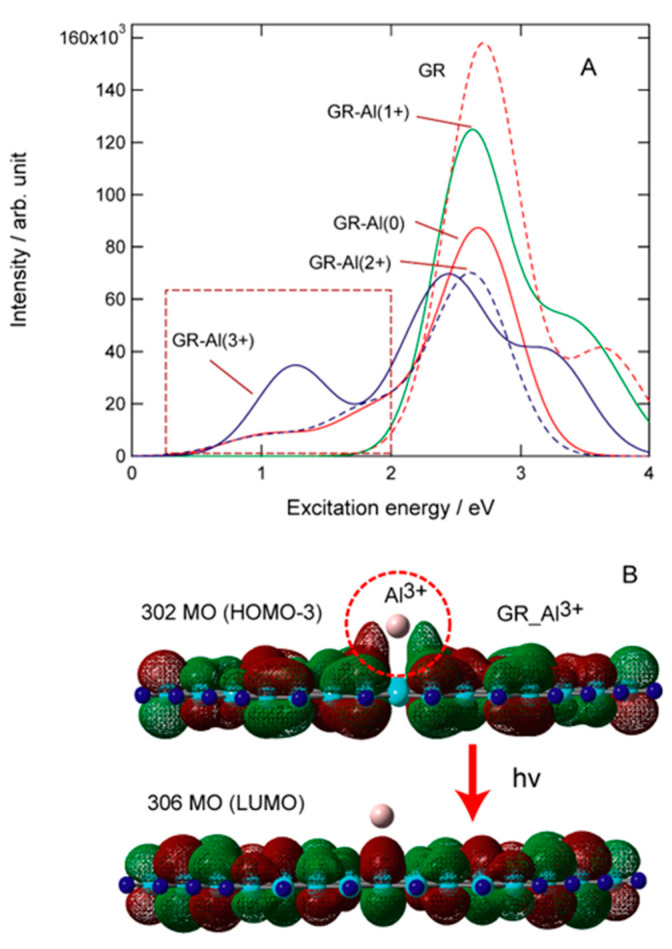
(**A**) Simulated absorption spectra of GR37 (without Al), GR-Al, GR-Al^+^, GR-Al^2+^, GR-Al^3+^ calculated at the CAM-B3LYP/6-31G(d) level, and (**B**) spatial distributions of MOs contributing to the absorption band appearing at 0.2–2.0 eV (low-energy bands) in GR37-Al^3+^.

Simulated absorption spectra of GR (without Al), GR-Al, GR-Al^+^, GR-Al^2+^, GR-Al^3+^ are given in Figure 2A. The calculations were carried out at the CAM-B3LYP/6-31G(d) level with GR37. The spectrum of GR (without Al) showed that the peak is located at E_ex_ = 2.70 eV, where E_ex_ means excitation energy. There was no absorption band in the ranges of 0.20–2.00 eV (hatched region: low-energy region). In GR-Al^+^, a single peak spectrum (E_ex_ = 2.60 eV) was obtained as the UV band, as well as that of GR (without Al). In contrast, a low-energy band newly appeared in GR-Al^3+^ in the range 0.2–2.0 eV (the peak is located at 1.27 eV) in the hatched region, and GR-Al^3+^ has a UV band with a peak at 2.42 eV. In cases of GR-Al^2+^ and GR-Al, the shoulder bands appeared in the low-energy region (0.2–2.0 eV) with the peak of 1.17 eV. These results suggested that a new lower-energy band is generated by Al’s doping to GR, except for GR-Al^+^.

To assign the low-energy bands appearing at 0.2–2.0 eV, the molecular orbitals (MOs) were analyzed in detail. The spatial distribution of MOs contributing to the absorption band (low-energy bands) are illustrated in Figure 2B. The excitation comprised an electronic transition from (HOMO-3) to LUMO. The electron in HOMO-3 was distributed in both Al and GR due to the CT from GR to Al, whereas there was no electron on Al in LUMO. This result suggested that the lower-energy band is assigned to be an electron-transfer band from Al to GR. The UV band can be assigned to the π–π* transition in GR (i.e., electron-back donation from Al to GR).

### 3.3. Diffusion of Al on Graphene Surface

**Figure 3:**
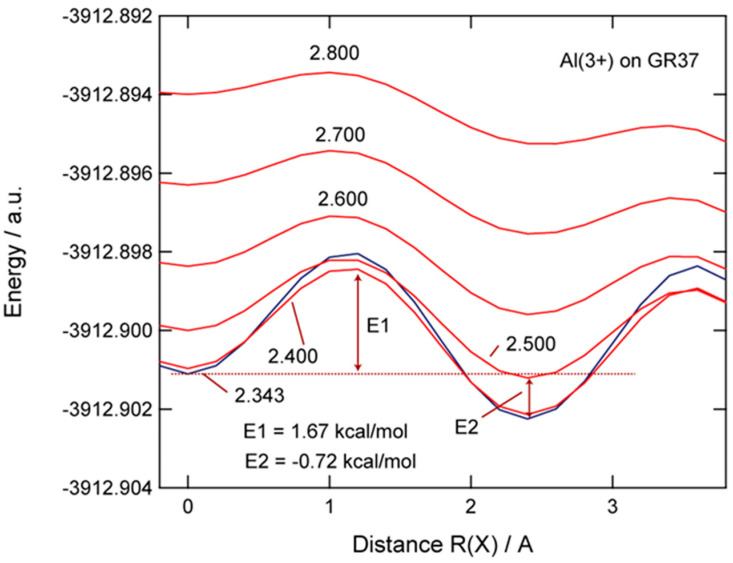
Potential energy curve (PECs) for the diffusion of Al^3+^ on GR37 surface (x-axis direction). The values mean height of Al from GR (h in Å).

In this section, the diffusion mechanism of Al on GR is discussed. Two diffusion paths were examined on the graphene surface, as shown in Figure 1. One is a diffusion path where Al moves along the x axis. First, Al was located in the hexagonal site (a). R(X) means Al’s distance from the hexagonal site (see Figure 1). The Al species can move from one hexagonal site to the next hexagonal site (b) via the center of the C-C bond of the benzene ring. The diffusion path is expressed as hexagonal site (a) → C-C bond center → hexagonal site (b). The other one is a diffusion path where Al moves along the y-axis and C-C bond line of the benzene ring. The diffusion path is expressed as hexagonal-site (a) → C-C bond line → hexagonal-site (c). The diffusion barrier for the y-axis was larger in energy than that for the x-axis. The potential energy curves of diffusion of Al^3+^ on GR are given in Figure 3. The heights of Al^3+^ from GR were selected as *h* = 2.342–2.800 Å, and the position of Al^3+^ was charged along the x-axis. It was found that a potential barrier exists in the center of the C-C bond of the benzene ring. Potential energy curves of diffusion of Al, Al^+^, and Al^2+^ on GR are given in Figures S1–S3 in the Supporting Information.

The diffusion barrier was calculated to be 1.67 kcal/mol for Al^3+^. Similar calculations were carried out for the y-axis direction. The y-axis energies were significantly higher than those for the x-axis, indicating that the diffusion along the x-axis direction is energetically favorable in GR-Al^3+^.

Similar calculations were carried out for the diffusion of Al and Al^+^, and Al^2+^ on the GR surface. The barrier heights are summarized in Table 3, together with those of Li^+^ and Na^+^ on GR. The diffusion barriers for Al and Al^+^, Al^2+^ and Al^3+^ were 2.44, 2.23, 1.53, and 1.67 kcal/mol, respectively. In contrast, the diffusion barriers of Li^+^ and Na^+^ on GR were 7.17 and 2.75 kcal/mol, respectively. These results strongly suggest that Al species diffusion is easy on the GR surface.

### 3.4. Structures of Molecular Hydrogen Bound to GR-Al

**Figure 4:**
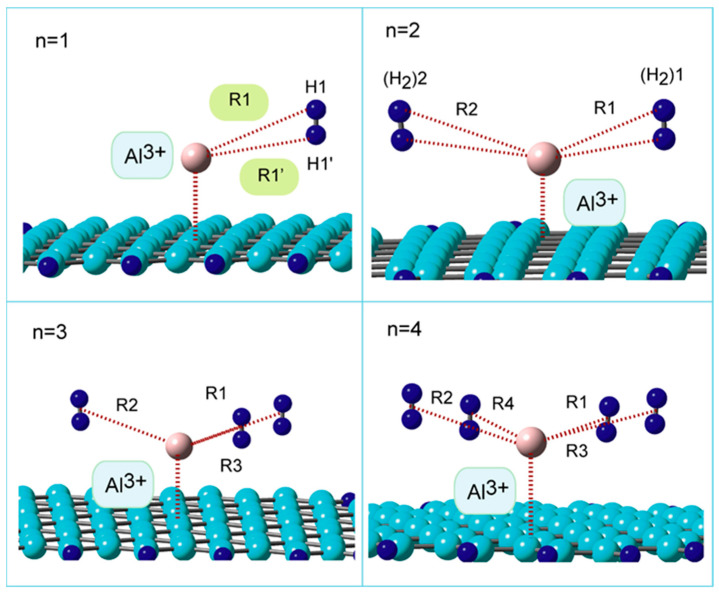
Optimized structures of GR19-Al^3+^-(H_2_)_n_ (*n* = 1–4). The calculations were performed at the CAM-B3LYP/6-31G(d) level.

The geometries of the GR-Al^3+^-(H_2_)_n_ systems (*n* = 1–8) were fully optimized at the CAM-B3LYP/6-31G(d) level. Figure 4 shows the structures of H_2_ bound to GR-Al (*n* = 1–4). In *n* = 1, H_2_ is bound to Al with a side-on structure, in which the two hydrogen atoms of H_2_ are almost equivalently connected to Al (distances are almost identical: R1 = 2.445, R1′ = 2.415 Å). The binding H_2_ molecule has an angle conformation: the angle of GR-Al- H_2_ was about 110 degrees. This conformation is due to the fact that H_2_ gains more binding energy from GR. The binding energy of H_2_ to Al was significantly small. H_2_ also interacts with the GR surface to increase the binding energy as much as possible. Hence, it takes on an angular conformation.

The interaction between the Al ion and H_2_ was composed of charge resonance interactions. Positive charges are resonantly transferred between Al and H_2_. The electron of the sigma-orbital of H_2_ donates to GR-Al^3+^. The binding of H_2_ is caused by the charge resonance effects.

The second H_2_ molecule, (H_2_)2, is bound to Al with a similar side-on arrangement in *n* = 2; (H_2_)1 and (H_2_)2 are bound in a similar manner to each other. The distances were R1 = 3.594 Å and R2 = 3.513 Å. The third and fourth H_2_ molecules are similarly bound to Al when *n* = 3 and 4. Similar binding structures were obtained for *n* = 5–8.

**Figure 5:**
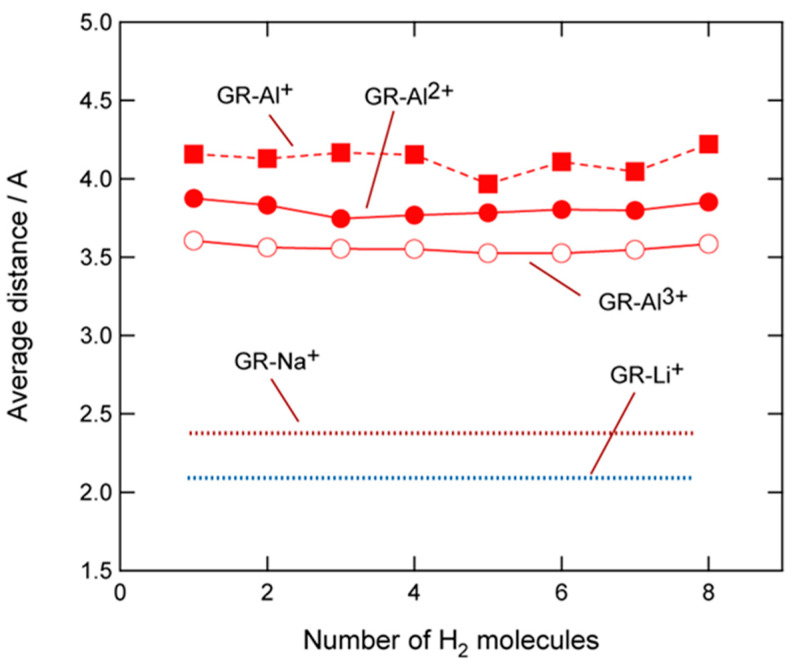
The average distances from Al to (H_2_)_n_, plotted as a function of n.

The average distances from Al to (H_2_)_n_ are plotted in Figure 5 as a function of *n*. The distances were calculated to be 2.262, 2.436, 2.456, and 2.502 Å for *n* = 1–4, respectively, which indicates that the distance increases slightly with an increasing number of H_2_ molecules (*n*).

The distances were almost constant (3.53–3.60 Å) in GR-Al^3+^. In Al^+^ and Al^2+^, the distances were longer than those of GR-Al^3+^: 3.75–3.85 Å for GR-Al^2+^ and 4.05–4.22 Å for GR-Al^+^. For comparison, the average distances of GR-Li^+^ and GR-Na^+^ are also plotted in Figure 5: 2.15 Å (Li^+^) and 2.47 Å (Na^+^). These values were significantly shorter than those of GR-Al. The interaction of the binding nature in the M = Li^+^ and Na^+^ systems is very different from that of GR-Al. In the former systems (Li^+^ and Na^+^), the ion-induced dipole interaction is dominant. In contrast, van der Waals (vdW) interaction is the main force in GR-Al.

### 3.5. H_2_-Binding Energies to GR-Al

**Figure 6:**
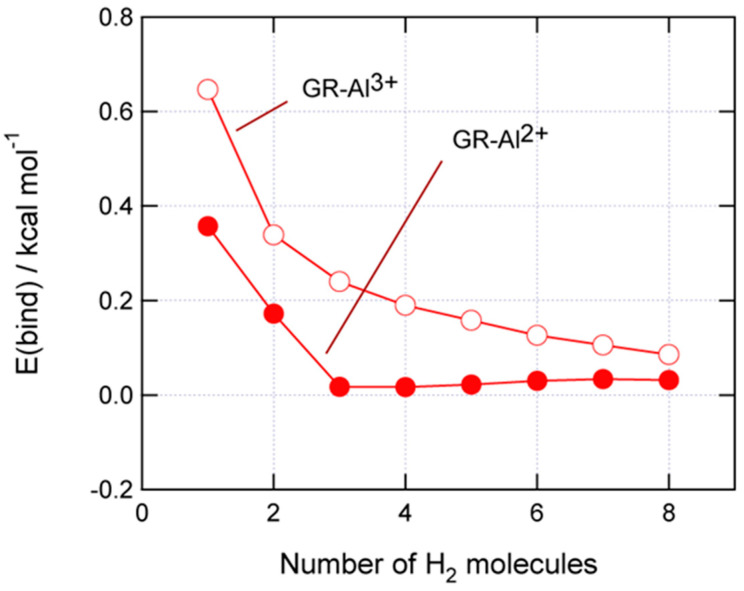
Binding energies of H_2_ to GR19-Al^3+^ and GR19-Al^2+^ (per H_2_ molecule).

The binding energies of H_2_ to GR-Al^3+^ and GR-Al^2+^ (per H_2_ molecule) are plotted in Figure 6 as a function of *n*. In *n* = 1, the binding energies were 0.65 (Al^3+^) and 0.36 (Al^2+^) kcal/mol. These values are significantly lower in energy than those of GR-Li^+^ (4.10 kcal/mol) and GR-Na^+^ (3.00 kcal/mol), suggesting that the H_2_-storage ability in GR-Al system is lower than those of the GR-Li^+^ and GR-Na^+^ systems. However, the binding energy between GR-Al^3+^ and H_2_ is larger than that of GR-H_2_ (less than 0.1 kcal/mol). As shown in Figure 6, the binding energies in GR-Al^3+^ monotonically decreased as a function of *n*: 0.24 kcal/mol (*n* = 3) and 0.09 kcal/mol (*n* = 8). In GR-Al^2+^, the binding energy was close to zero, larger than *n* = 3 (0.02 kcal/mol). These results indicate that the GR-Al^3+^ system can be used as a weak H_2_ storage material. Note that H_2_ was not bound in *n* = 9.

## 4. Conclusions

In the present study, the effects of Al doping to GR were investigated using the DFT method in order to design Al-functionalized graphene from a theoretical point of view. The calculations showed that Al species can adsorb on GR surface: the neutral, mono-, di-, and trivalent Al ion (Al, Al^+^, Al^2+^, and Al^3+^, respectively) were examined as the Al species. The binding nature is mainly a charge transfer between Al and GR. Al-doping to GR caused a new energy band originating from a charge transfer between Al and GR.

The diffusion path and energy barrier heights of Al on GR were determined for all Al species. The Al species diffuse from one hexagonal site to the next hexagonal site via the C-C bond center of benzene rings. The barrier heights were calculated to be 1.5–2.4 kcal/mol, and were slightly dependent on the charge of Al. These values are significantly lower in energy than that of Li^+^ on graphene (6.3–7.3 kcal/mol) [31]. These results suggested that Al species can be used as the materials for an ion battery composed of graphene. The binding energy of H_2_ to GR-Al^3+^ was calculated to be 0.7 kcal/mol, which are significantly lower than those of the other metal ions: 4.2 (Li^+^), 3.0 (Na^+^), 13.2 kcal/mol (Mg^2+^). However, the binding energy for 0.7 kcal/mol is larger than the interaction energy of H_2_ to GR (0.1 kcal/mol). Therefore, the GR-Al system can be used as a weak H_2_ storage system.

In the present study, we investigated the structures and electronic states of GR-Al. In addition, the hydrogen storage capacity of GR-Al was examined. The doping of Al ions to GR creates new electronic bands in low-energy regions. This indicates its potential as an electronic material. In addition, GR-Al weakly adsorbs H_2_ and could be used as an easy H_2_-desorption material. In near future, we plan to study the adsorption of NH_3_, CO_2_, and CO to elucidate its potential as a chemical sensor.

## Figures and Tables

**Table 1:** Heights of Al species from GR37 surface (*h* in Å), NPA charges on Al and GR37, and binding energies of Al to GR (E(bind) in kcal/mol). The calculations were carried out at the CAM-B3LYP/6-311G(d,p) level.

Al Species	*h*	NPA	NPA	*E*(*bind*)
		Al	GR37	
**Al**	2.217	+0.864	−0.864	1.9
**Al^+^**	2.273	+0.931	+0.069	45.3
**Al^2+^**	2.333	+0.924	+1.076	297.7
**Al^3+^**	2.406	+0.912	+2.088	730.5

**Table 2:** Heights of Al species from GR19 surface (*h* in Å), NPA charges on Al and GR19, and binding energies of Al to GR19 (E(bind) in kcal/mol). The calculations were carried out at the CAM-B3LYP/6-31G(d) level.

Al Species	*h*	NPA	NPA	*E*(*bind*)
		Al	GR19	
**Al**	2.195	+0.848	−0.848	0.8
**Al^+^**	2.240	+0.861	+0.139	47.8
**Al^2+^**	2.349	+0.885	+1.115	280.1
**Al^3+^**	2.515	+0.911	+2.089	679.5

**Table 3:** Diffusion barriers of GR37-Al and GR37-M (M = Li^+^ and Na^+^) (E(diffuse) in kcal/mol).

Al Species	E(diffuse)	M	E(diffuse)
**Al**	2.44	Li^+^	7.17
**Al^+^**	2.23	Na^+^	2.75
**Al^2+^**	1.53	-	-
**Al^3+^**	1.67	-	-

## Data Availability

Not applicable.

## Supplementary Materials

The following supporting information can be downloaded at: https://www.mdpi.com/article/10.3390/nano13142046/s1, Figure S1: Potential energy curve (PECs) for the diffusion of Al on GR37 surface (x-axis direction). The values mean height of Al from GR (*h* in Å); Figure S2: Potential energy curve (PECs) for the diffusion of Al^3^ on GR37 surface (x-axis direction). The values mean height of Al from GR (*h* in Å); Figure S3: Potential energy curve (PECs) for the diffusion of Al^2+^ on GR37 surface (x-axis direction). The values mean height of Al from GR (*h* in Å).
