# Supplementary material for: Aluminum-Doping Effects on the Electronic States of Graphene Nanoflake: Diffusion and Hydrogen Storage Mechanism
Source: Nanomaterials (Basel). 2023 Jul 11;13(14):2046. doi: 10.3390/nano13142046 (PMC10384847; doi:10.3390/nano13142046)
Supplement: Supplementary file 1 [file nanomaterials-13-02046-s001.zip › nanomaterials-2491517-supplementary.pdf]

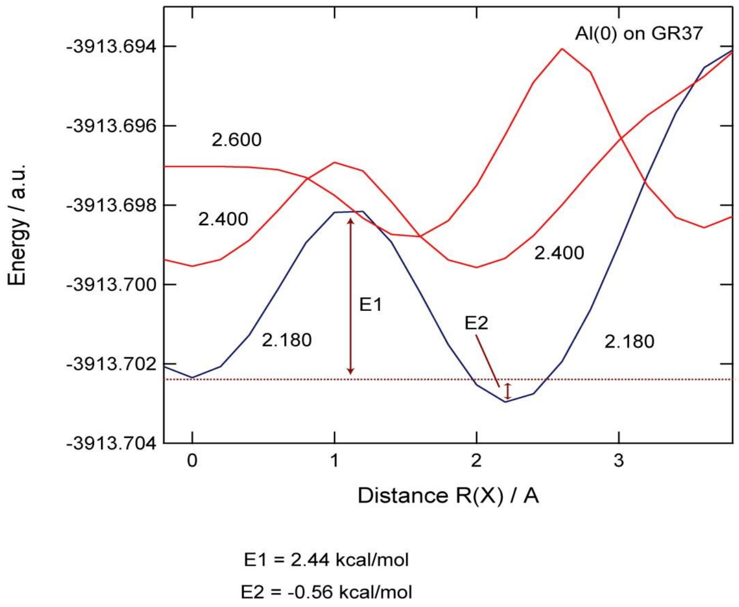

Figure S1. Potential energy curve (PECs) for the diffusion of Al on GR37 surface (x-axis direction). The values mean height of Al from GR (*h* in Å).

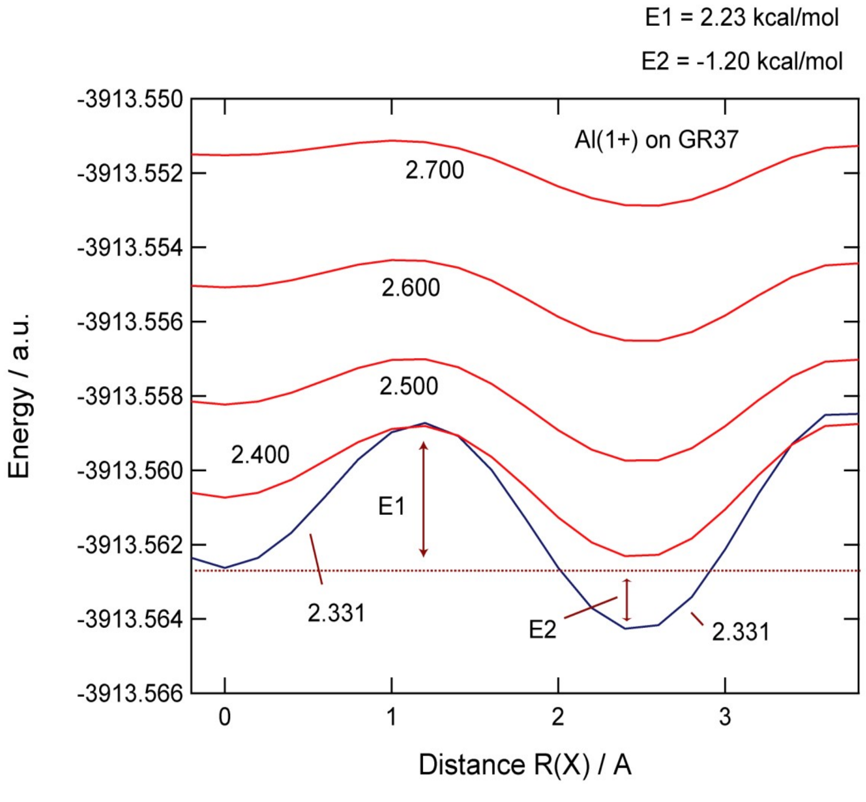

Figure S2. Potential energy curve (PECs) for the diffusion of Al[3] on GR37 surface (x-axis direction). The values mean height of Al from GR (*h* in Å). .

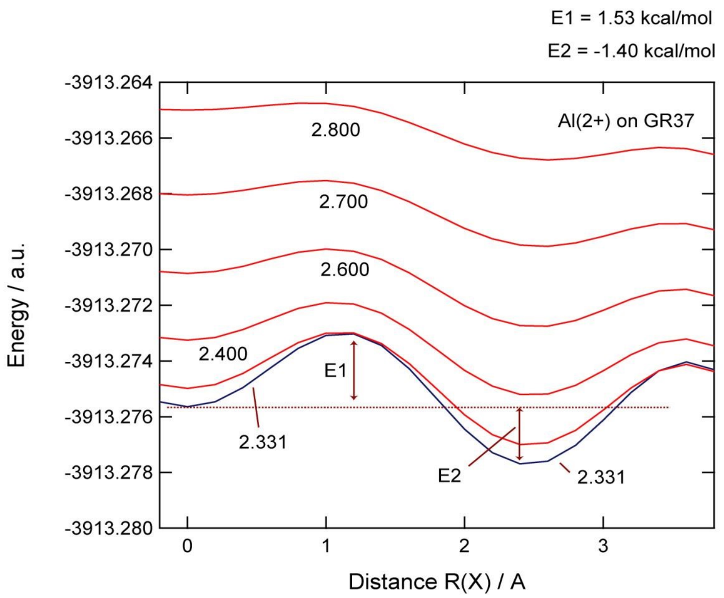

Figure S3. Potential energy curve (PECs) for the diffusion of Al^{2+} on GR37 surface (x-axis direction). The values mean height of Al from GR (h in Å). .
